# Therapeutic Potential of Nitrogen Mustard Based Hybrid Molecules

**DOI:** 10.3389/fphar.2018.01453

**Published:** 2018-12-17

**Authors:** Yiming Chen, Yuping Jia, Weiguo Song, Lei Zhang

**Affiliations:** ^1^Department of Medicinal Chemistry, School of Pharmacy, Weifang Medical University, Weifang, China; ^2^Shandong Academy of Pharmaceutical Science, Jinan, China

**Keywords:** antitumor, nitrogen mustard, hybrids, side effects, drug discovery

## Abstract

As medicine advances, cancer is still among one of the major health problems, posing significant threats to human health. New anticancer agents features with novel scaffolds and/or unique mechanisms of action are highly desirable for the treatment of cancers, especially those highly aggressive and drug-resistant ones. Nitrogen mustard has been widely used as an anticancer drug since the discovery of its antitumor effect in the 1942. However, the lack of selectivity to cancer cells restricts the wide usage of a mass of nitrogen mustard agents to achieve further clinical significance. Discovery of antitumor hybrids using nitrogen mustards as key functional groups has exhibited enormous potential in the drug development. Introduction of nitrogen mustards resulted in improvement in the activity, selectivity, targetability, safety, pharmacokinetics and pharmacodynamics properties of corresponding lead compounds or agents. Herein, the recently developed nitrogen mustard based hybrids have been introduced in the cancer therapy.

## Introduction

**FIGURE 1:**
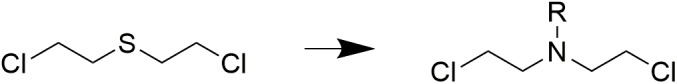
Origin of nitrogen mustards.

In recent years, malignant tumors have become a serious threat to human health due to their worldwide rising incidence and mortality. Second to cardiovascular diseases, cancer contributed the second most mortalities among all diseases (Torre et al., 2015; Ryerson et al., 2016; Lallukka et al., 2017). In recent decades, development of antitumor drugs has achieved significant progress in the treatment of cancer. Since nitrogen mustard, known as an alkylating agent, was proven effective in the treatment of malignant lymphoma in the 1940s, the usage of nitrogen mustard drugs in cancer chemotherapy has a history of over 70 years. At present, nitrogen mustard agents are still used clinically, and targeted modification of nitrogen mustards is an important strategy for the discovery of anticancer drugs. The development of nitrogen mustard derivatives originated from bis(2-chloroethyl) sulfide, which was used as a poison gas during World War II (Gilman, 1963; DeVita and Chu, 2008). After a terrible accident, it was found that bis(2-chloroethyl) sulfide exhibited therapeutic potential on leukemia. Because of its severe toxicity, bis(2-chloroethyl) sulfide was not applied as a antitumor drug for clinical use. However, nitrogen mustard antitumor drugs were developed based on the leukocyte killing effect of bis(2-chloroethyl) sulfide (Figure 1).

Nitrogen mustard is a kind of bio-alkylating agent, which can form active electron-deficient intermediates or other compounds with active electrophilic groups *in vivo*. The active intermediates can react electrophilically with some electron-rich groups in bio-macromolecules by forming covalent bonds, and results in activity inhibition of corresponding bio-macromolecules. The mechanism of nitrogen mustards includes DNA binding and cross-linking, thus preventing DNA replication and cell proliferation. Since its binding to the N7 nitrogen-atoms on DNA guanines with poor selectivity, nitrogen mustard agents are revealed to be toxic to normal cells (Kohn et al., 1987; Bank et al., 1989; Povirk and Shuker, 1994; Di Antonio et al., 2014).

Clinical application of nitrogen mustard compounds has a long history, but the present and future application of nitrogen mustards is limited by disadvantages including poor selectivity and severe adverse reactions (Frei et al., 1988; Sanderson and Shield, 1996; Schobert et al., 2009; Chen et al., 2014). Therefore, enormous effort has been made in the development of nitrogen mustard derivatives, aiming to obtain antitumor nitrogen mustard drugs with high activity and low toxicity (Zarytova et al., 1990). In recent years, the discovery of nitrogen mustard drugs and derivatives has become attractive field in the anticancer therapy. Development of nitrogen mustard based hybrid molecules by introducing druggable fragment, has been considered to be effective strategy in the antitumor drug discovery. Herein, recently development of nitrogen mustard based hybrids was reviewed and provided suggestions for the future study of bifunctional and multitargeted antitumor drugs.

## Nitrogen Mustard Drugs

According to different carriers, nitrogen mustard drugs can be classified into several classes, including fatty nitrogen mustard, aromatic nitrogen mustard, amide nitrogen mustard, amino acid and polypeptide nitrogen mustard, and heterocyclic nitrogen mustard.

**FIGURE 2:**
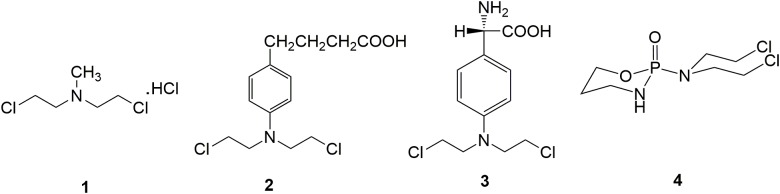
Representative nitrogen mustard agents.

Chlormethine **1** (Figure 2), a fatty nitrogen mustard, is now rarely used for clinic due to its poor selectivity and severe toxicity. The introduction of aromatic rings into nitrogen mustard causes the decrease of electrophilicity of the nitrogen atom. Consequently, aromatic nitrogen mustards are characterized with reduced reactivity and toxicity compared with fatty nitrogen mustard (Goodman and Wintrobe, 1946). Chlorambucil **2** (Figure 2) is used clinically for the treatment of ovarian cancer, Hodgkin’s disease, chronic lymphocytic leukemia and lymphosarcoma (Galton et al., 1961). Clinical application of chlorambucil is also limited by adverse effects including nausea, vomiting, anemia, bone marrow suppression and neurotoxicity (Springer et al., 1990; Nicolle et al., 2004). Melphalan **3** (Figure 2), which takes phenylalanine as the carrier, has exhibited clinical effects on ovarian cancer, breast cancer, lymphoid sarcoma and multiple myeloma (Sarosy et al., 1988). Cyclophosphamide **4** (Figure 2), a heterocyclic amide nitrogen mustard, features a board spectrum of anti-malignancy activity, and is commonly utilized in the management of malignant lymphoma, acute lymphoblastic leukemia, multiple myeloma, lung cancer, neuroblastoma, breast cancer, ovarian cancer and nasopharyngeal cancer (Hughes et al., 2018). Moreover, cyclophosphamide has been discovered to be less toxicity than other types of nitrogen mustard drugs, due to the specific metabolic pathway.

## Nitrogen Mustard Based Hybrids

In recent years, it has been revealed that the conjugation of targeted antitumor drugs or natural molecules with nitrogen mustard drugs provides novel strategies for the discovery of antitumor molecules with improved antitumor effect, selectivity, and reduced toxicity.

**FIGURE 3:**
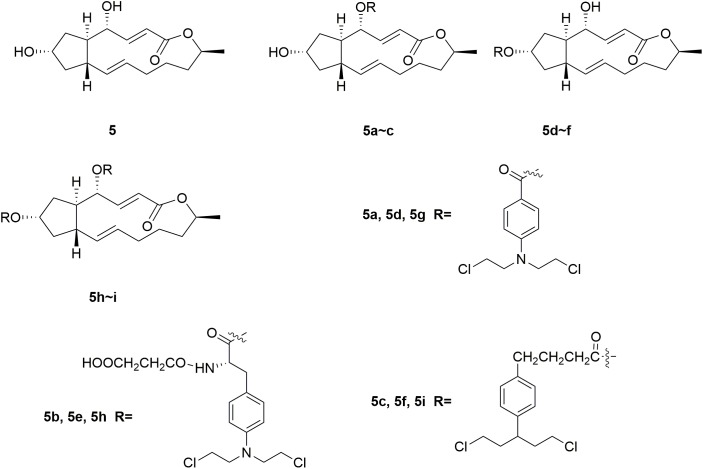
Structures of Brefeldin A-nitrogen mustard hybrids.

Brefeldin A (BFA) **5** (Figure 3) is a 16-member macrolide antibiotic with a broad range of pharmacological activities, including antitumor, antiviral and antifungal effects (Rajamahanty et al., 2010; Moon et al., 2012; South et al., 2013; Toda et al., 2015; Grose and Klionsky, 2016; Huang et al., 2017). In the antiproliferative activity assay, BFA exhibited GI_50_ (half maximal growth inhibitory concentration) value of 40 nM against the national cancer institute NCI-60 cancer cell line (Anadu et al., 2006). Although BFA has great potentials to serve as a cancer chemotherapeutic drug, its development is still restricted by major limitations including severe undesirable effects and relatively low selectivity on tumor cells over normal ones (Kikuchi et al., 2003; Seehafer et al., 2013). Several novel BFA-nitrogen mustard conjugates were derived by introducing nitrogen mustards at 4-OH and/or 7-OH of BFA (Han et al., 2018). All the synthesized BFA-nitrogen mustard compounds **5a-i** (Figure 3) were assessed for their effectiveness against different tumor cell lines. Several hybrid molecules exhibited potent cytostatic activities and improved selectivity on malignant cells over normal ones. It is revealed that almost all the new BFA-nitrogen mustards showed stronger cytotoxic activities against one or more cell lines than nitrogen mustards and even 5-FU. Among all the tested compounds, molecule **5a** exhibited the most potent antiproliferative effects against various tumor cell lines (with IC_50_ (half maximal inhibitory concentration) values of 4.48, 9.37, 0.2, and 0.84 μM against human leukemia HL-60, human prostate PC-3, human hepatocellular carcinoma Bel-7402 and drug-resistant Bel-7402/5-FU cell lines), respectively. Molecule **5a** also displayed much lower cytotoxicity (IC_50_ < 0.001 μM) than BFA (IC_50_= 9.74 μM) against normal human hepatic L-O2 cells. Therefore, introduction of nitrogen mustard to toxic natural products could be significant in the improvement the potency and safety of lead compounds.

**FIGURE 4:**
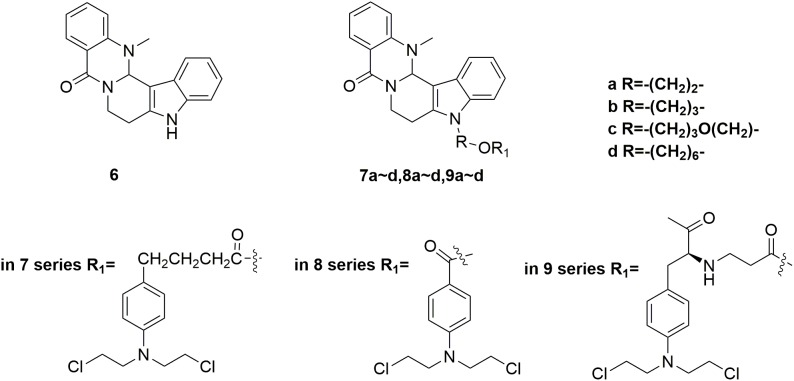
Structures of evodiamine-nitrogen mustard hybrids.

Evodiamine **6** (Figure 4) is a natural quinolone alkaloid widely studied for the treatment of diverse human disorders including Alzheimer’s disease, inflammation and especially cancer (Ogasawara et al., 2002; Yu et al., 2013; Lv et al., 2015; Shi L. et al., 2016; Wang et al., 2016; Wu et al., 2016; Fan et al., 2017; Shi et al., 2017). By targeting topoisomerase I and II, evodiamine has induced apoptosis and cell cycle arrest of a broad spectrum of tumor cell lines (Shyu et al., 2006). However, it is revealed that evodiamine is cytotoxic to human normal cells, such as peripheral blood mononuclear cells (PBMC). In discovery of antitumor agent with improved potency and reduced adverse side effects, conjunct of evodiamine to nitrogen mustards was carried out by Li and coworkers (Hu et al., 2017). The synthesized nitrogen mustard-evodiamine hybrids were evaluated in the antitumor activity assay. Compared with evodiamine (IC_50_ values of 22.87 μM against PBMC cells), all the tested mustard-evodiamine hybrids **7a-d, 8a-d, 9a-d** (Figure 4) showed improved safety properties with IC_50_ values of more than 200 μM in inhibition the proliferation of PBMC cells. Remarkably, molecule **9c** revealed potent antiproliferative effects against human liver cancer HepG2, human leukemic THP-1 and HL-60 cell lines with IC_50_ value of 17.04 μM, 4.05 μM and 0.50 μM, respectively. The involved investigations indicated that further drug discovery based on **9c** is promising in the treatment of tumor, such as leukemia. Collectively, introduction of nitrogen mustard moiety has shown significance in the improvement of potency and safety, and the nitrogen mustard hybridation strategy could be productive for the optimization of lead compounds.

**FIGURE 5:**
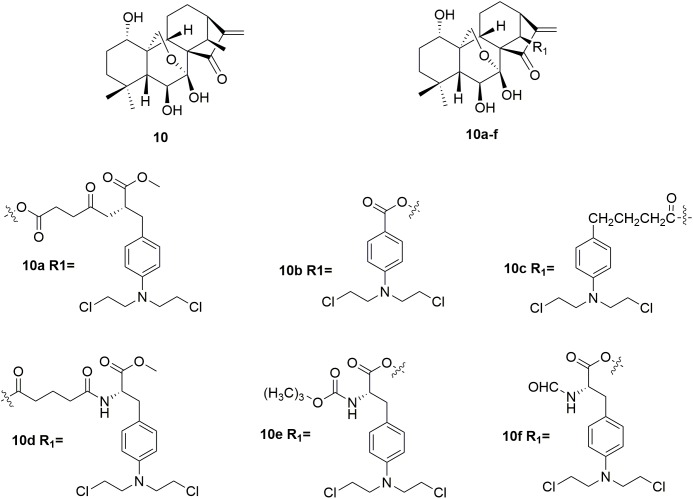
Structures of oridonin-nitrogen mustard hybrids.

Oridonin **10** (Figure 5) is a kind of natural diterpenoids, which has a unique, safe, broad antitumor activity (Sun et al., 2006; Cui et al., 2007; Zhou et al., 2007; Bao et al., 2014; Li Y. et al., 2015; Ding et al., 2016; Dong et al., 2016; Shi M. et al., 2016; Liu et al., 2017). However, the utilization of oridonin in cancer chemotherapy was limited by its relatively low potency (Wang et al., 2012; Xu S.T. et al., 2014). Development of oridonin-nitrogen mustard conjugates used for antitumor application has been demonstrated to be promising in the drug discovery (Ding et al., 2013a,b). Several synthetic oridonin-nitrogen mustard conjugates **10a∼f** (Figure 5), and their anticancer activities evaluated in four human malignant cell lines (human leukemia K562 cells, human breast cancer MCF-7 cells, human hepatocellular carcinoma Bel-7402 cells, and human gastric cancer MCG-803 cells) were reported by Xu and coworkers (Xu S. et al., 2014). All the tested compounds exhibited better antiproliferative effects comparing to the positive control drugs, melphalan, chlorambucil and benzoic acid mustard. Among the synthetic oridonin mustards, compound **10b** was the most potent hybrid against MCF-7 and Bel-7402 cells with IC_50_ values of 0.68 μM and 0.50 μM, respectively. It is also revealed that **10b** and **10c** could inhibit the growth of drug-resistant cancer cells. Notably, molecule **10b** exhibits approximately eight-fold higher selectivity for cancer cells over normal cells, which is significantly higher than its parent oridonin compound and clinically available nitrogen mustard drugs. Collectively, the derived oridonin-nitrogen mustard conjugates exhibited improved activity and safety than the parent fragments, and introduction of nitrogen mustard make contributions to the potency and selectivity of oridonin based hybrids.

**FIGURE 6:**
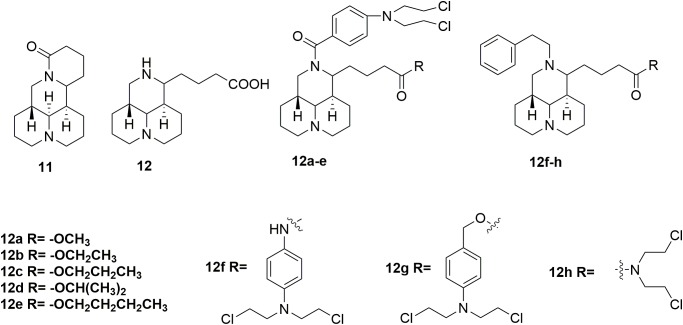
Structures of sophoridine-nitrogen mustard hybrids.

In addition to evodiamine, another alkaloid, sophoridine **11** (Figure 6) evaluated in detailed for its antitumor potency, was approved by the CFDA in 2005 for treatment several types of cancer, including liver, gastric and lung cancer (Sun et al., 2012; Liu and Liu, 2013; Wang et al., 2014). The sophoridine which could cause apoptotic cell death by inhibiting DNA topoisomerase I activity and initiate cell cycle arrest at the G0/G1 phase, has high solubility and good safety profiles (Quo et al., 2013). However, the moderate anticancer activity of sophoridine limit its clinical application. Therefore, development of sophoridine derivatives was performed in discovery of more effective drug candidates. The D-ring of sophridine has been opened to generate sophoridinic acid **12** (Figure 6) for further structural modification. A series of sophoridinic acid-nitrogen mustard deivatives **12a-h** (Figure 6) were derived by modifying 12 nitrogen atom and carboxyl groups of **12** (Li D.D. et al., 2015). Compared with sophoridine (IC_50_ > 80 μM against human liver cancer HepG2 cells), several new synthesized hybrids showed improved antitumor activity. Especially compound **12f** showed IC_50_ value of 0.47 μM compared with melphalan (IC_50_ value of 0.41 μM) in the inhibition of HepG2 cells. SAR analysis indicated two promising substituents on the 12-nitrogen atom and carboxyl region, which were helpful for maintaining potent antitumor activity. Moreover, various decorating various substituents may be introduced to these two moieties, regulating the pharmacological effects of the compounds. Introduction of the cyclophosphamide metabolite (phosphamide mustard A) analogs also resulted in hybrids with significantly improved activities compared with sophoridine (Li D. et al., 2018). It is demonstrated that the introduction of nitrogen mustard on sophoridine could significantly improve interactions between sophoridine and DNA-Topo I, and subsequently increase the antitumor activity. Therefore, the study of nitrogen mustard as the parent drug is of great significance in the design and synthesis of antitumor drugs.

**FIGURE 7:**
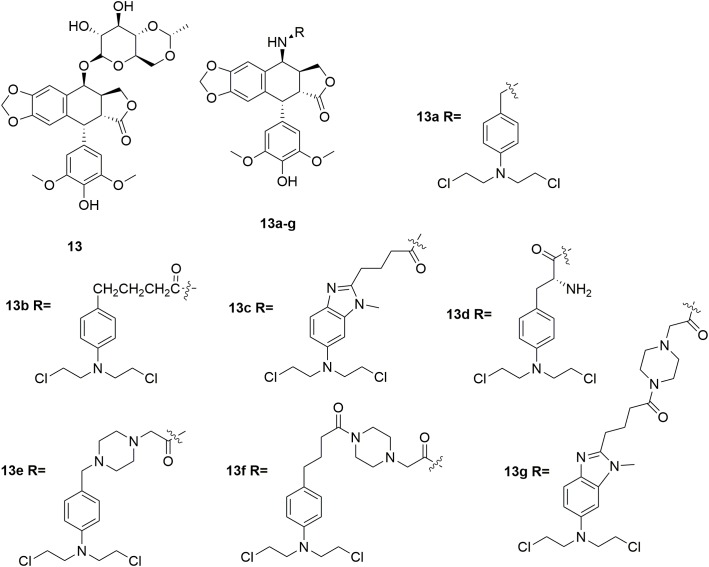
Structures of etoposide-nitrogen mustard hybrids.

Etoposide **13** (Figure 7) is a topoisomerase II inhibitor effective in the treatments of various types of cancer including testicular cancer, lung cancer, lymphoma, leukemia, neuroblastoma, and ovarian cancer (Nitiss, 2009; Pommier and Marchand, 2011; Pommier, 2013). In discovery of etoposide analogs, glycoside moiety of etoposide was replaced by nitrogen mustard moiety designed to alkylate either protein residues on topoisomerase II, or the DNA bases on the DNA-topoisomeraseII complex (Deweese and Osheroff, 2009; Pommier et al., 2010; Wu et al., 2011). Seven N-mustard–epipodophyllotoxin hybrid compounds **13a-g** (Figure 7) were synthesized, and demonstrated to target topoisomerase II by kDNA decatenation assay, DNA cleavage assay, cellular ICE assay and the cell cycle analyses (Yadav et al., 2014). The derived molecules also exhibited nitrogen mustard-alike activity as it crosslinked DNA. In the *in vitro* antiproliferative assay, molecule **13e** exhibited the best antiproliferative activity with IC_50_ values of 0.27 μM and 0.85 μM against human leukemia K562 cells and etoposide-resistant K/VP.5 cells, and GI_50_ of 0.71 μM against NCI-60 cells in contrast to the control melphalan (IC_50_ values of 12 μM and 5.3 μM against K562 cells and K/VP.5 cells, and GI_50_ of 29 μM against NCI-60 cells) and etoposide (IC_50_ values of 0.29 μM and 4.9 μM against K562 cells and K/VP.5 cells, and GI_50_ of 12 μM against NCI-60 cells). The results suggested that hybridization of etoposide and nitrogen mustards is promising in the development of highly potent antitumor molecules both by topoisomerase II inhibition as well as DNA alkylation.

**FIGURE 8:**
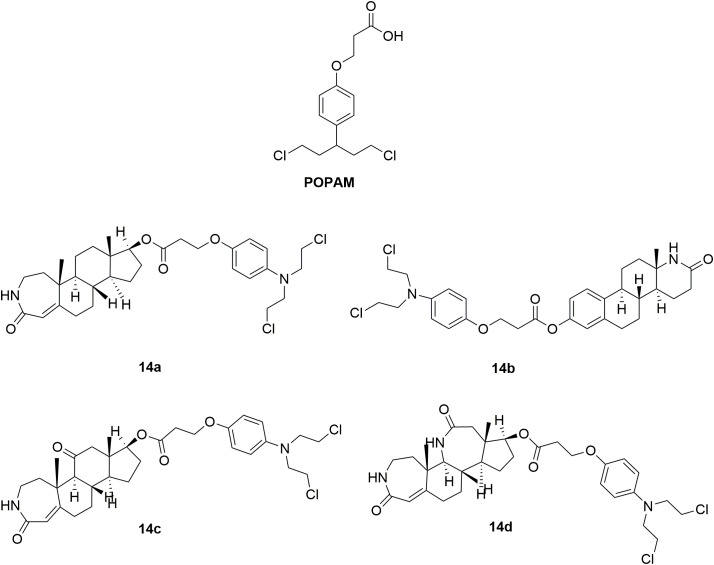
Structures of steroid-nitrogen mustard hybrids.

In order to decrease toxicity of nitrogen mustards, steroids have been tested as a vehicle to deliver the mustard drugs to a specific target tissue via interaction with steroid receptors (Wall et al., 1969; Catane, 1978). Such conjugates improved the lipophilicity and solubility of the resulting drugs. Development of steroidal alkylating agents has been reviewed by Bérubé and coworkers (Trafalis et al., 2016). Herein, the recently derived novel steroidal lactam derivatives and 3-(4-(bis(2-chloroethyl)amino)phenoxy)propanoic acid (POPAM) (Figure 8) conjunctions were described (Trafalis et al., 2016). Four new ester conjugates **14a-d** (Figure 8) of steroidal lactams with POPAM were synthesized and tested against human leukemia cell lines *in vitro*. Molecule **14c** was discovered to be the most potent hybrid with IC_50_ values of 90 μM, 65 μM, 80 μM, and 85 μM against human leukemia MOLT-4, CCRF-CEM, JURKAT and SUP-B15 cells compared with melphalan (IC_50_ > 100 μM against all the test cell lines) and POPAM (IC_50_ > 100 μM against all the test cell lines), respectively. In the *in vivo* antiproliferative assay, **14c** also exhibited improved antileukemic activity compared with their alkylating component alone (POPAM). Moreover, in the *in vivo* acute toxicity test, all the derived hybrids had significantly lower acute toxicity (LD_10_ (10% lethal dose) > 80 mg/kg), in contrast to the non-steroidal alkylators POPAM (LD_10_ = 14 mg/kg) and melphalan (LD_10_ = 15 mg/kg). Further investigation revealed that the chemical linkage between the nitrogen mustard and the lactam-steroids seems to both decreased the toxicities of the nitrogen mustards and improved the bioactivity and antitumor effects.

**FIGURE 9:**
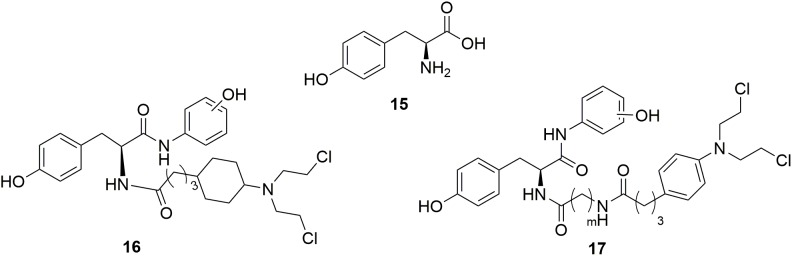
Structures of Tyrosine-nitrogen mustard hybrids.

Tyrosine **15** (Figure 9), a natural amino acid, has been reported to share some structural similarities to that of the phenol group of estradiol (Anstead et al., 1997). Molecular modeling study indicated that the phenol group of tyrosine also interact with the estrogen receptor binding site in the same manner as the A-ring phenol of estradiol. Therefore, the tyrosine was modified to mimic the structure of estradiol (Muthyala et al., 2003; Descoteaux et al., 2012b). Tyrosinamide, combined tyrosine with hydroxyaniline, was proved to be structurally similar to estradiol. A series of tyrosinamide-nitrogen mustard derivatives were synthesized and tested by Bérubé and coworkers (Descoteaux et al., 2012a). It is revealed that all new compounds showed potent antitumor activities. Among the derived tyrosinamide-chlorambucil hybrids, compound ***m*-16** (Figure 9), showed IC_50_ values of 48.61 and 31.25 μM against human breast cancer MDA-MB-231 cells and MCF-7 cells compared with the parent compounds chloramucil (IC_50_ values of 136.85 and 130.36 μM against MDA-MB-231 cell and MCF-7 cells), respectively. Compared with chloramucil (IC_50_ values of 63.17, 66.11, 100.48 and 131.83 μM against human ovarian carcinoma A2780 cells, OVCAR-3 cells, human breast cancer ZR-75-1 cells and MDA-MB-468 cells, respectively), the ***m*-17** (Figure 9) showed potent antitumor activity with IC50 values of 31.79, 35.42, and 52.10 μM against OVCAR-3 cells, MDA-MB-468 cells and ZR-75-1 cells, respectively. It is found that all the synthesized tyrosinamide-chlrambucil molecules exhibited improved inhibitory activity in the inhibition of breast, ovarian and uterine cancer cells than the parental chlorambucil. Introduction of tyrosine entity to nitrogen mustards was considered to make contributes to the increased antitumor activity of the derived hybrid molecules.

**FIGURE 10:**
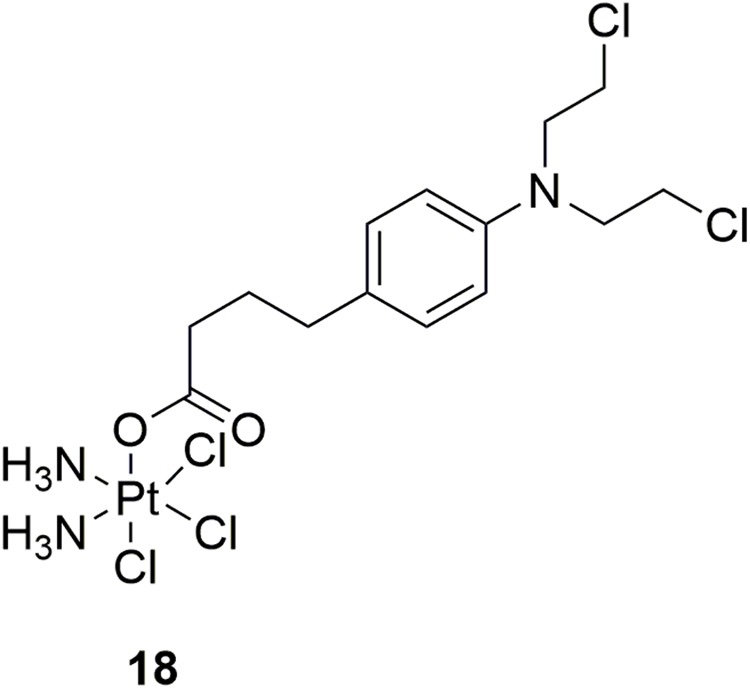
Structure of Platinum-nitrogen mustard hybrid.

Platinum-based antineoplastic drugs are usually considered as another class of alkylating agents with high antitumor potency (Jamieson and Lippard, 1999; Wang and Lippard, 2005). Notably, cisplatin is one of the most potent platinum(II) complexes used in cancer chemotherapy by binding to DNA and subsequently interfere with replication and transcription, eventually leading to cellular apoptosis (Abu-Surrah and Kettunen, 2006; Wheate et al., 2010). However, clinical application of cisplatin is limited by its severe adverse effects, including nephrotoxicity, hepatotoxicity, ototoxicity and neurotoxicity, etc. Acquired resistance is also a concern (Brabec et al., 2017). In discovery of more potent and safe antitumor compounds, conjunction of two different types of DNA-damaging drugs by combining chlorambucil with platinum(IV) complexes was performed by Gou and coworkers (Qin et al., 2017). In the *in vitro* activity assay, molecule **18** (Figure 10), a hybrid of cisplatin and chlorambucil, displayed potent antiproliferative activities with IC_50_ values of 3.99 μM, 4.37 μM, 4.97 μM, 2.97 μM, and 4.23 μM against human breast cancer MCF-7, human colon cancer HCT-116, human liver cancer HepG-2, human gastric cancer SGC7901, and cisplatin-resistant SGC7901/CDDP cells, respectively. Compared with chlorambucil, cisplatin, and oxaliplatin, molecule **18** exhibited improved activity in the inhibition of cisplatin resistant SGC7901/CDDP cells. Further studies revealed that molecule **18** induced cell cycle arrest at S/G2 phases (distinct from those of cisplatin and chlorambucil), and revealed ability of overcome drug resistance. Collectively, hybridization of nitrogen mustards and platinum(IV) complexes resulted in conjunctions with improved antitumor potency, and with advantage of overcoming drug resistance of tumor cells.

**FIGURE 11:**
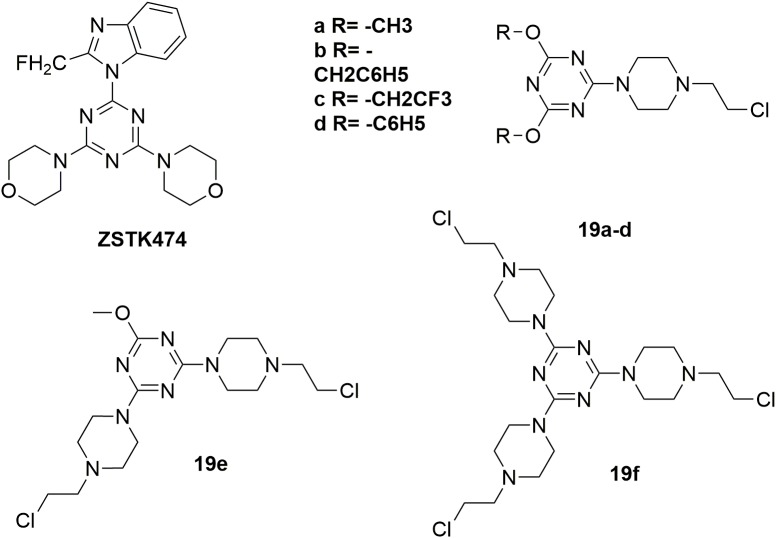
Structures of melamine-nitrogen mustard hybrids.

Several highly potent 1,3,5-triazine scaffold-carrying cytostatic agents have been previously reported as inhibitors of cell proliferation-involved enzymes (Maeda et al., 2000; Riou et al., 2002; Gomez et al., 2003; Kaminski et al., 2004; Mandal et al., 2007; Paquin et al., 2008). Among them, ZSTK474 (Figure 11) was discovered to inhibit the growth of tumor cells in human cancer xenografts without toxic effects on critical organs by targeting PI3K (Di Francesco et al., 2000; Vedejs et al., 2003). In the structural modification of current melamine derivatives, a series of melamine-nitrogen mustard derivatives **19a-f** (Figure 11) were synthesized by introducing one or more 2-chloroethylamine groups (Kolesinska et al., 2012). It is revealed that all synthesized molecules showed potent antitumor activities. Compared with the positive control chlorambucil (IC_50_ value of 29.14 μM against human breast cancer MCF-7 cells), the obtained molecule **19f** showed potent antitumor activity with IC_50_ value of 18.70 μM against MCF-7 cells. Compound **19a** also exhibited potent antitumor activities with IC_50_ value of 0.62 μM, 0.99 μM, 1.40 μM, 2.06 μM and 3.45 μM against human leukemia Jurkat, human prostate adenocarcinoma LNCaP, human breast adenocarcinoma T47D, human lung adenocarcinoma A549 and human colorectal carcinoma SW707 cells, respectively. Further biological studies suggested that introducing nitrogen mustard into triazine is promising in the increase of antitumor activity by promoting alkylation. Accordingly, introduction of nitrogen mustard is regarded to make contribution to improve the selectivity, activity and lipophilicity of current drug-like cytotoxic derivatives.

**FIGURE 12:**
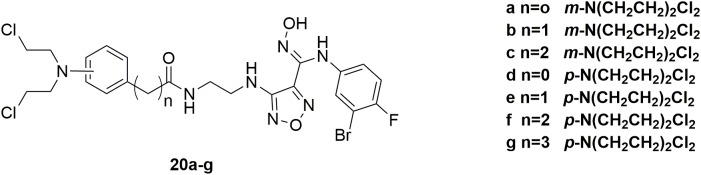
Structures of IDO1-nitrogen mustard hybrids.

IDO1, a heme-containing enzyme, plays an important role in carcinogenesis and its progression by converting Trp to Kyn (Jiang et al., 2015). Both Trp degradation and Kyn accumulation are associated with immune tolerance by affecting T cell activity and altering the tumor microenvironment (Moon et al., 2015). A number of studies showed that the combination of IDO1 inhibitors along with cytotoxic chemotherapeutic agents is an effective strategy in cancer treatment (Muller et al., 2005). However, such a simple combination will inevitably be limited by the severe adverse effects induced by the highly toxic cytotoxic agents and possible drug-drug interactions. Therefore, in discovery of potent antitumor molecules with reduced toxicity, a series of hybrid molecules **20a-g** (Figure 12) were synthesized by including the pharmacophores of both IDO1 inhibitors and nitrogen mustards (Fang et al., 2018). All the compounds showed potent antitumor activities compared with the positive drug chlorambucil in the inhibition of murine colorectal carcinoma CT-26, human lung adenocarcinoma A549, human colon cancer HCT116 and human colorectal adenocarcinoma HT-29 cells. Obviously, compound **20a** significantly inhibited IDO1 activity in tumor tissues and reduced Kyn level in plasma with IDO1 inhibitory IC_50_ value of 0.13 μM and antiproliferative EC_50_ (half maximal effect concentration) value of 0.27 μM aganist HeLa cells. Moreover, molecule **20a** exhibited high potent *in vivo* antitumor efficacy (tumor growth inhibition (TGI) = 58.2%) compared with clinical candidate IDO1 inhibitor epacadostat (TGI = 47.5%) in the allograft animal model with CT-26 without remarkable body weight loss or adverse effects. In conclusion, it is revealed that introduction of nitrogen mustard into pharmacophores of IDO1 inhibitors could significantly improve the antitumor activity and reduce toxicity of parent compound in the antitumor evaluation.

## Conclusion and Perspective

Nitrogen mustards represent the earliest studied DNA cross-linking agents, and application of DNA alkylating agents has been widely utilized for the treatment of cancer for more than 70 years. In spite of their long history, several nitrogen mustard drugs, including cyclophosphamide, chlorambucil, and melphalan, still remained as first line antitumor agents in the management of various types of tumors. However, the clinical application of nitrogen mustards was restricted by their undesired adverse effects, relatively low efficacy compared with targeted antitumor drugs, and drug resistance caused by enhanced drug inactivation, decreased cellular uptake, enhanced DNA repair and/or DNA damage tolerance. It is generally accepted that cancer has its pathological root in genetical mutations, affecting cell replication. Thus, targeting different proliferative mechanisms by the construction of hybrid anticancer drugs seem to be a promising strategy. Development of nitrogen mustard based hybrids has been revealed to be effective strategy in discovery of antitumor drugs with increased activity, reduced toxicity, and improved physicochemical properties such as the lipophilicity and the solubility.

**FIGURE 13:**
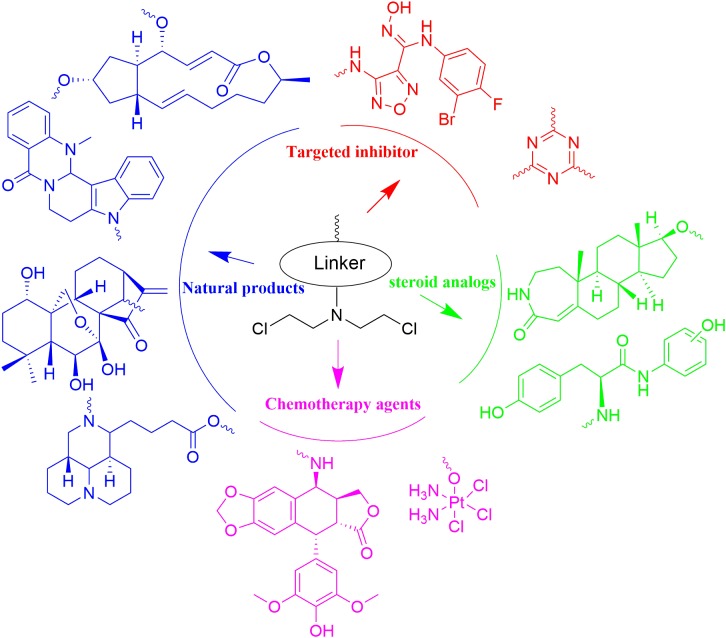
Summary of the nitrogen mustard based hybridization strategy involved in the current article.

With N,N-bis(2-chloroethyl)amine as functional group, nitrogen mustards has been hybridized with various drug-like fragments (Figure 13). Novel hybrids have been derived with improved potency, selectivity, safety, pharmacokinetics, pharmacodynamics properties and/or broader range of therapeutic activities. Current achievements make development of nitrogen mustard based conjunctions to be attractive area in the cancer treatment. Despite the reported advantages, unexpected side effects caused by introduction of nitrogen mustards also require careful attention in the drug design and biological evaluation. As questioned on the efficacy of twin drugs and prodrugs, it is also necessary to demonstrate advantages of the conjugates linked by ester bond or more stable bonds in comparison with the combined therapy with parental drugs.

## Author Contributions

YC and YJ participated in most of the literature retrieval and article writing. LZ and WS guided the review and revised the writing.

## Conflict of Interest Statement

The authors declare that the research was conducted in the absence of any commercial or financial relationships that could be construed as a potential conflict of interest.
